# Extracellular purines in lung endothelial permeability and pulmonary diseases

**DOI:** 10.3389/fphys.2024.1450673

**Published:** 2024-08-20

**Authors:** Evgenia Gerasimovskaya, Rahul S. Patil, Adrian Davies, McKenzie E. Maloney, Liselle Simon, Basmah Mohamed, Mary Cherian-Shaw, Alexander D. Verin

**Affiliations:** ^1^ Department of Pediatrics, University of Colorado Denver, Aurora, CO, United States; ^2^ Vascular Biology Center, Medical College of Georgia, Augusta University, Augusta, GA, United States; ^3^ Department of Internal Medicine, Medical College of Georgia, Augusta University, Augusta, GA, United States; ^4^ Office of Academic Affairs, Medical College of Georgia, Augusta University, Augusta, GA, United States; ^5^ Department of Medicine, Medical College of Georgia, Augusta University, Augusta, GA, United States

**Keywords:** extracellular purines, purinergic receptors, endothelial cells, endothelial barrier function, vasa vasorum, angiogenesis, pulmonary diseases

## Abstract

The purinergic signaling system is an evolutionarily conserved and critical regulatory circuit that maintains homeostatic balance across various organ systems and cell types by providing compensatory responses to diverse pathologies. Despite cardiovascular diseases taking a leading position in human morbidity and mortality worldwide, pulmonary diseases represent significant health concerns as well. The endothelium of both pulmonary and systemic circulation (bronchial vessels) plays a pivotal role in maintaining lung tissue homeostasis by providing an active barrier and modulating adhesion and infiltration of inflammatory cells. However, investigations into purinergic regulation of lung endothelium have remained limited, despite widespread recognition of the role of extracellular nucleotides and adenosine in hypoxic, inflammatory, and immune responses within the pulmonary microenvironment. In this review, we provide an overview of the basic aspects of purinergic signaling in vascular endothelium and highlight recent studies focusing on pulmonary microvascular endothelial cells and endothelial cells from the pulmonary artery vasa vasorum. Through this compilation of research findings, we aim to shed light on the emerging insights into the purinergic modulation of pulmonary endothelial function and its implications for lung health and disease.

## 1 Introduction

Extracellular purines, including adenosine triphosphate (ATP), adenosine diphosphate (ADP), adenosine monophosphate (AMP), and adenosine, are versatile signaling molecules that exert their effects in a cell- and tissue-specific manner. Acting through purinergic receptors, specifically P1 (A1, A2A, A2B, and A3) for adenosine and P2 (P2X and P2Y) for ATP, ADP, and UTP, purines regulate a wide array of physiological processes in health and disease (Abbracchio et al., 2006; Jacobson and Gao, 2006; Burnstock, 2007; Erlinge and Burnstock, 2008; Fredholm et al., 2011; Jacobson et al., 2012; Sheth et al., 2014). The extensive expression of purinergic receptors underscores the critical role of purinergic signaling in maintaining homeostatic balance across various organ systems, whether in states of health or disease. Essential components of this signaling system include ATP release, extracellular hydrolysis leading to adenosine production, and subsequent cellular uptake of adenosine.

The endothelium, forming the inner lining of blood vessels, plays a crucial role in regulating vascular permeability, maintaining homeostasis, and orchestrating immune responses within the lungs. Vascular endothelial cells, abundant in purinergic receptors, are particularly sensitive to purinergic regulation, exhibiting diverse responses such as pro-angiogenic, adhesive, pro-inflammatory, and barrier-protective actions. Endothelial cells from different vascular beds possess tissue-specific characteristics, contributing to the complexity of purinergic signaling in vascular physiology (Aird, 2007a; Aird, 2007b). The lung vasculature encompasses two distinct types of vessels: the pulmonary and systemic (bronchial) circulation (Herve and Fadel, 2006; Tuder et al., 2007; McCullagh et al., 2010; Galambos et al., 2016; Jia et al., 2023). The bronchial circulation extends its influence to large extrapulmonary vessels, including the main pulmonary artery and the right and left pulmonary arteries, forming the vasa vasorum network within the vascular wall’s adventitial and medial compartments (Stenmark et al., 2013; Humbert et al., 2019; Phillippi, 2022). Endothelial cells from both pulmonary and systemic circulation significantly influence the development of various lung diseases, ranging from acute respiratory distress syndrome (ARDS), chronic obstructive pulmonary disease (COPD), emphysema, idiopathic pulmonary fibrosis (IPF), pulmonary hypertension, lung cancer, acute lung injury (ALI) via the dysregulation of endothelial barrier function, angiogenic, and adhesive properties (Carmeliet, 2003; Voelkel and Cool, 2003; Lucas et al., 2009; Goh et al., 2011; Sakao et al., 2014; Huertas et al., 2018; Vassiliou et al., 2020; May et al., 2023).

Despite the association between these lung diseases and elevated extracellular nucleotide levels, there is a scarcity of studies elucidating the role of purinergic signaling in pathological endothelial responses. In this review, we present evidence suggesting that in pulmonary endothelium, extracellular ATP and adenosine mediate barrier-protective responses. Conversely, in pulmonary artery vasa vasorum endothelial cells (bronchial circulation), ATP and ADP promote angiogenesis, while adenosine exerts a barrier-protective effect. Therefore, through this review, we aim to shed light on some previously unrecognized features of purine signaling in barrier protective and angiogenic mechanisms in vascular endothelium.

## 2 Extracellular purines as important regulators of vascular functions

**FIGURE 1:**
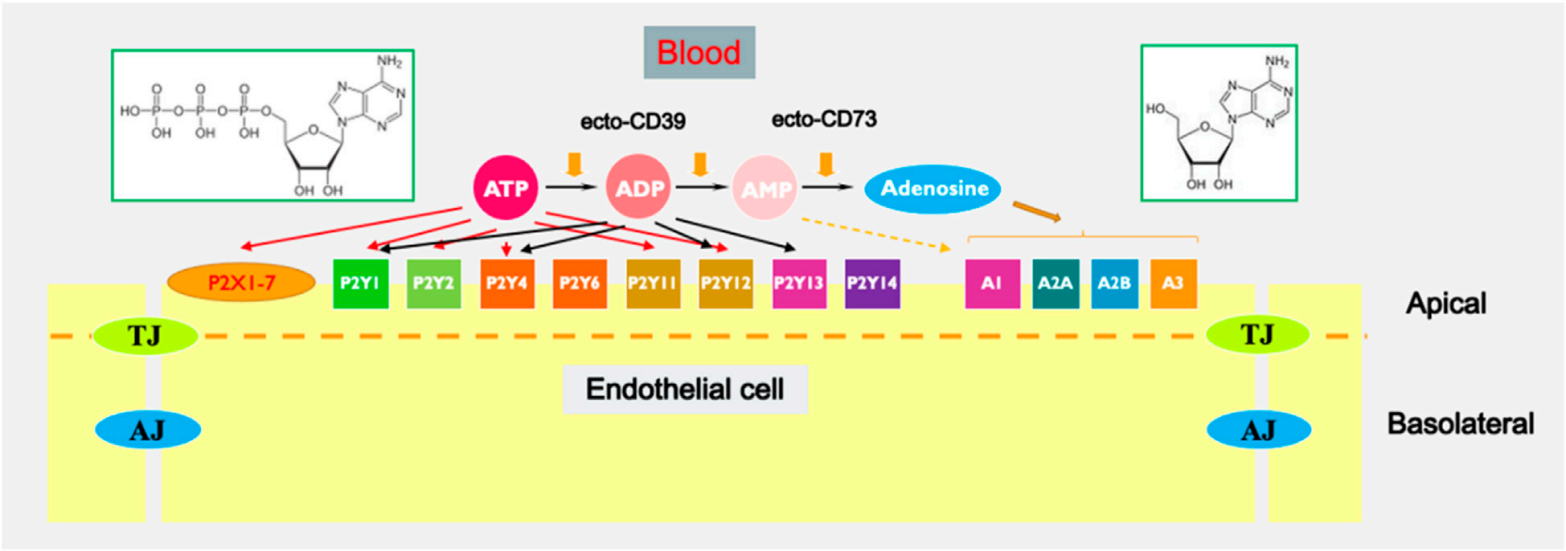
Extracellular purine-mediated signaling in endothelium. Chemical structures of ATP (left) and adenosine (right) are shown in green-boarded rectangles.

Extracellular purines and pyrimidines, including ATP, ADP, AMP, adenosine, UTP, and UDP, have been recognized as significant regulators of various cellular processes in the vasculature. These processes include proliferation, migration, chemotaxis, and inflammatory responses, which are associated with a range of cardiovascular and pulmonary diseases (Di Virgilio and Solini, 2002; Bours et al., 2006; Burnstock, 2006; Yegutkin, 2008; Burnstock, 2009; Burnstock and Ralevic, 2013; Kanthi et al., 2014; Burnstock and Pelleg, 2015; Visovatti et al., 2016; Burnstock, 2017a; Cai et al., 2020; Wirsching et al., 2020). Extracellular nucleotides commonly exert their effects by interacting with two distinct classes of purinergic receptors: P2Y receptors (metabotropic) and P2X receptors (ionotropic) (Abbracchio et al., 2006; Jacobson and Gao, 2006; Burnstock, 2007; Erlinge and Burnstock, 2008; Fredholm et al., 2011; Jacobson et al., 2012; Sheth et al., 2014). Conversely, adenosine functions via P1 receptors, comprising A1, A2A, A2B, and A3 subtypes, to exert its effects. While P2X receptors serve as ATP-gated ion channels, whereas P1 and P2Y receptors are linked to heterotrimeric G-proteins (Figure 1) (Table 1).

**TABLE 1:** Extracellular purines, their receptors and main signaling.

P1	A1	Adenosine	Go, Gi, Gβγ/AC/cAMP ↓; Gq/PLC/Ca^2+^ + PKC ↑	Murthy and Makhlouf (1995), Tomura et al. (1997), Cordeaux et al. (2004), Tawfik et al. (2005), Schepp and Reutershan (2008), Yang et al. (2015), Borea et al. (2018)
	A2A	Adenosine	Go, Gs/AC/cAMP ↑	Iwamoto et al. (1994), Németh et al. (2003), Schepp and Reutershan (2008), Sun and Huang (2016), Borea et al. (2018)
	A2B	Adenosine	Gs/cAMP ↑; Gq/PLC/Ca^2+^ + PKC ↑	Iwamoto et al. (1994), Schepp and Reutershan (2008), Sun and Huang (2016), Borea et al. (2018)
	A3	Adenosine	Gi/AC/cAMP ↓; Gq/PLC/Ca^2+^ + PKC ↑	Palmer et al. (1995), Zhao et al. (2000), Schepp and Reutershan (2008), Kanno et al. (2012), Borea et al. (2018)
P2	P2X1-P2X7	ATP	Ligand-gated cation channels	Coddou et al. (2011), Kaczmarek-Hájek et al. (2012)
	P2Y1	ADP/ATP	Gq/PLC/Ca^2+^ + PKC ↑; ERK1/2↑	Communi et al. (1997), Murthy and Makhlouf (1998), Communi et al. (1999), Filippov et al. (1999), Vanhaesebroeck and Waterfield (1999), Jiang et al. (2000), Guba et al. (2002), Filippov et al. (2004), Waldo and Harden (2004), Abbracchio et al. (2006), Wullschleger et al. (2006), Gerasimovskaya et al. (2008), Morello et al. (2009), Ryzhov et al. (2009), Umapathy et al. (2010a), Carr et al. (2010), Lyubchenko et al. (2011), Zemskov et al. (2011), Nguyen Dinh Cat et al. (2013), Díaz-Vegas et al. (2015), Wang et al. (2016)
	P2Y2	ATP/UTP	Gq/PLC/Ca^2+^ + PKC ↑	Communi et al. (1997), Murthy and Makhlouf (1998), Communi et al. (1999), Filippov et al. (1999), Filippov et al. (2004), Waldo and Harden (2004), Abbracchio et al. (2006), da Silva et al. (2009), Ryzhov et al. (2009), Umapathy et al. (2010a), Nguyen Dinh Cat et al. (2013), Eun et al. (2014), Díaz-Vegas et al. (2015), Wang et al. (2015), Agca et al. (2016), Wang et al. (2016), Feliu et al. (2019), Strassheim et al. (2020a)
	P2Y4	ATP/ADP	Gq/PLC/Ca^2+^ + PKC↑; Gi/AC/cAMP ↓	Communi et al. (1997), Murthy and Makhlouf (1998), Filippov et al. (1999), Suarez-Huerta et al. (2001), Filippov et al. (2004), Waldo and Harden (2004), Kolosova et al. (2005), Abbracchio et al. (2006), Erlinge and Burnstock (2008), da Silva et al. (2009), Ryzhov et al. (2009), Umapathy et al. (2010a), Zemskov et al. (2011), Nguyen Dinh Cat et al. (2013), Díaz-Vegas et al. (2015), Wang et al. (2016), Bátori et al. (2019)
	P2Y6	UDP	Gq/PLC/Ca^2+^ + PKC ↑	Communi et al. (1997), Murthy and Makhlouf (1998), Communi et al. (1999), Filippov et al. (1999), Filippov et al. (2004), Waldo and Harden (2004), Ryzhov et al. (2009), Umapathy et al. (2010a), Nguyen Dinh Cat et al. (2013), Díaz-Vegas et al. (2015), Wang et al. (2016)
	P2Y11	ATP	Gq/PLC/Ca^2+^ ↑ + PKC; Gs/AC/cAMP ↑	Communi et al. (1997), Murthy and Makhlouf (1998), Communi et al. (1999), Filippov et al. (1999), van der Weyden et al. (2000), Qi et al. (2001), Klinger et al. (2002), Filippov et al. (2004), Waldo and Harden (2004), Yaar et al. (2005), Zylberg et al. (2007), Ryzhov et al. (2009), Umapathy et al. (2010a), Zemskov et al. (2011), Dănilă et al. (2020)
	P2Y12	ATP/ADP	Gi/AC/cAMP ↓	Kolosova et al. (2005), Abbracchio et al. (2006), Zhan et al. (2007), Bátori et al. (2019)
	P2Y13	ADP	Gi/AC/cAMP ↓; Gq/PLC/Ca^2+^ ↑; ERK1/2↑	Marteau et al. (2003), Abbracchio et al. (2006), Lyubchenko et al. (2011), Zhang et al. (2019)
	P2Y14	UDP/UDP-Glucose	Gi/AC/cAMP ↓	Abbracchio et al. (2006), Carter et al. (2009), Fricks et al. (2009)

The widespread presence of purinergic receptors across different tissues, coupled with the expression of multiple receptor subtypes in vascular cells, underscores the physiological significance of the purinergic signaling system in regulating vascular cell function. Additionally, there is growing recognition that changes in purinergic signaling physiology can contribute to the onset and progression of various diseases, including cancer, neurodegenerative disorders, immune dysregulation, and infectious diseases (Bours et al., 2006; Burnstock, 2006; Abbracchio et al., 2009; Antonioli et al., 2013; Idzko et al., 2014; Burnstock, 2017a; Burnstock, 2017b; Di Virgilio et al., 2017; Ferrari et al., 2018; Vultaggio-Poma et al., 2020). In light of this understanding, targeting purinergic receptors with specific ligands, whether agonists or antagonists, emerges as a promising strategy for selectively modulating pathway-specific intravascular responses associated with different pathological conditions (Ohta and Sitkovsky, 2009; Gessi et al., 2011; Müller and Jacobson, 2011; Jacobson and Müller, 2016; Strassheim et al., 2020b; Mühleder et al., 2020; Wernly and Zhou, 2020; Wirsching et al., 2020).

### 2.1 Extracellular ATP and vascular diseases

Multiple studies provided evidence suggesting that extracellular nucleotides may play a role in the pathogenesis of vascular diseases. For instance, extracellular ATP has been linked to arterial wall hyperplasia and hypertrophy in spontaneously hypertensive animals (Ralevic and Burnstock, 1998; Burnstock, 2017a; Cai et al., 2020). Additionally, ATP has been implicated in vascular inflammation and endothelial dysfunction observed in atherosclerosis (Stachon et al., 2016; Wu et al., 2023), as well as in the pathophysiology of respiratory and infectious diseases (Idzko et al., 2007; Riteau et al., 2010; Burnstock et al., 2012; Wirsching et al., 2020). Purinergic signaling plays a pivotal role in distal lungs, regulating vascular remodeling, inflammation, and contractility in pulmonary hypertension (Erlinge and Burnstock, 2008; Visovatti et al., 2016; Cai et al., 2020), as well as in the regulation of vascular permeability (Noll et al., 1999; Kolosova et al., 2005; Jacobson et al., 2006; Strassheim et al., 2020b; Aslam et al., 2021; Maier-Begandt et al., 2021), proliferative and migratory responses of vascular endothelial, hematopoietic, and resident stem cells (Satterwhite et al., 1999; Lemoli et al., 2004; Kaczmarek et al., 2005; Rossi et al., 2007; Niimi et al., 2017; Ma et al., 2023).

The stimulation of DNA synthesis in adventitial fibroblasts, smooth muscle cells (SMCs), and vascular endothelial cells has been demonstrated upon exposure to extracellular UTP and ATP (Van Daele et al., 1992; Erlinge, 1998; Gerasimovskaya et al., 2002; Gerasimovskaya et al., 2008; Strassheim et al., 2020a). Extracellular nucleotides have also been observed to directly influence the migration of both vascular and non-vascular cells, such as monocytes and neutrophils (Kaufmann et al., 2005; Kukulski et al., 2007). Studies in the literature have shown that in endothelial cells (EC), stimulation of P2Y2 receptors by UTP and ATP is associated with the co-activation of VEGF receptor-2, upregulation of Vascular Cell Adhesion Molecule-1 (VCAM-1), and recruitment of monocytes. These findings suggest a connection between purinergic signaling, angiogenesis, and inflammatory responses (Seye et al., 2003; Seye et al., 2004). In addition, adenosine, the product of ATP hydrolysis, has been demonstrated to elicit proliferative responses in diverse types of EC (Ethier et al., 1993; Feoktistov et al., 2004; Huertas et al., 2020).

Significantly, ATP collaborates with cytokines such as insulin-like growth factor, epidermal growth factor, and platelet-derived growth factor, along with integrins, to facilitate proliferation and migration of vascular cells. This underscores the physiological importance of extracellular ATP, particularly in hypoxic and stress-related contexts (Huang et al., 1989; Erlinge, 1998; Gerasimovskaya et al., 2002; Kaczmarek et al., 2005). Recent research has also uncovered the involvement of extracellular ATP and UTP in stimulating the migration of human hematopoietic stem cells both *in vitro* and *in vivo* (Lemoli et al., 2004; Rossi et al., 2007). These findings collectively underscore the angiogenic potential of extracellular nucleotides and their pivotal role in regulating vascular cell function across various physiological and pathological contexts, including hypoxia and inflammation. They also demonstrate the role of purinergic signaling in modulating hypoxia-induced vascular cell phenotypes.

### 2.2 Role of plasma membrane ecto-nucleotidases in a regulation purinergic signaling

Enzymes involved in the metabolism of extracellular nucleotides are important regulators of purinergic signaling (Robson et al., 2006; Yegutkin, 2008; Zimmermann et al., 2012; Yegutkin, 2014) (Figure 1). NTPDase1/CD39 plays a crucial role in modulating purinergic signaling by hydrolyzing extracellular ATP and ADP to AMP, which is further metabolized by CD73 to adenosine. This enzymatic activity of CD39 regulates the levels of extracellular ATP and ADP, thereby influencing purinergic signaling pathways involved in inflammation, immune responses, thrombosis, and tissue homeostasis. Several studies have demonstrated the therapeutic potential of targeting CD39 in various pathological conditions. In the context of cardiovascular diseases, CD39-mediated inhibition of platelet aggregation and thrombosis has been explored as a potential strategy to prevent arterial thrombosis and ischemic events. For example, NTPDase1/CD39 was shown to eliminate vascular thrombosis and inflammation (Kanthi et al., 2014; Covarrubias et al., 2016; Anyanwu et al., 2019; Yadav et al., 2019), whereas purinergic antithrombotic drugs demonstrated efficacy in lowering the likelihood of recurrent strokes and heart attacks (Burnstock, 2006). The pharmacological targeting of CD39 in vascular cells represents a promising therapeutic approach for modulating thrombosis, inflammation, and angiogenesis in various vascular-related diseases. Further research is needed to elucidate the precise mechanisms underlying CD39-mediated effects and to develop targeted therapies that effectively harness its therapeutic benefits.

### 2.3 Sources and stimuli of ATP release in the vasculature

**FIGURE 2:**
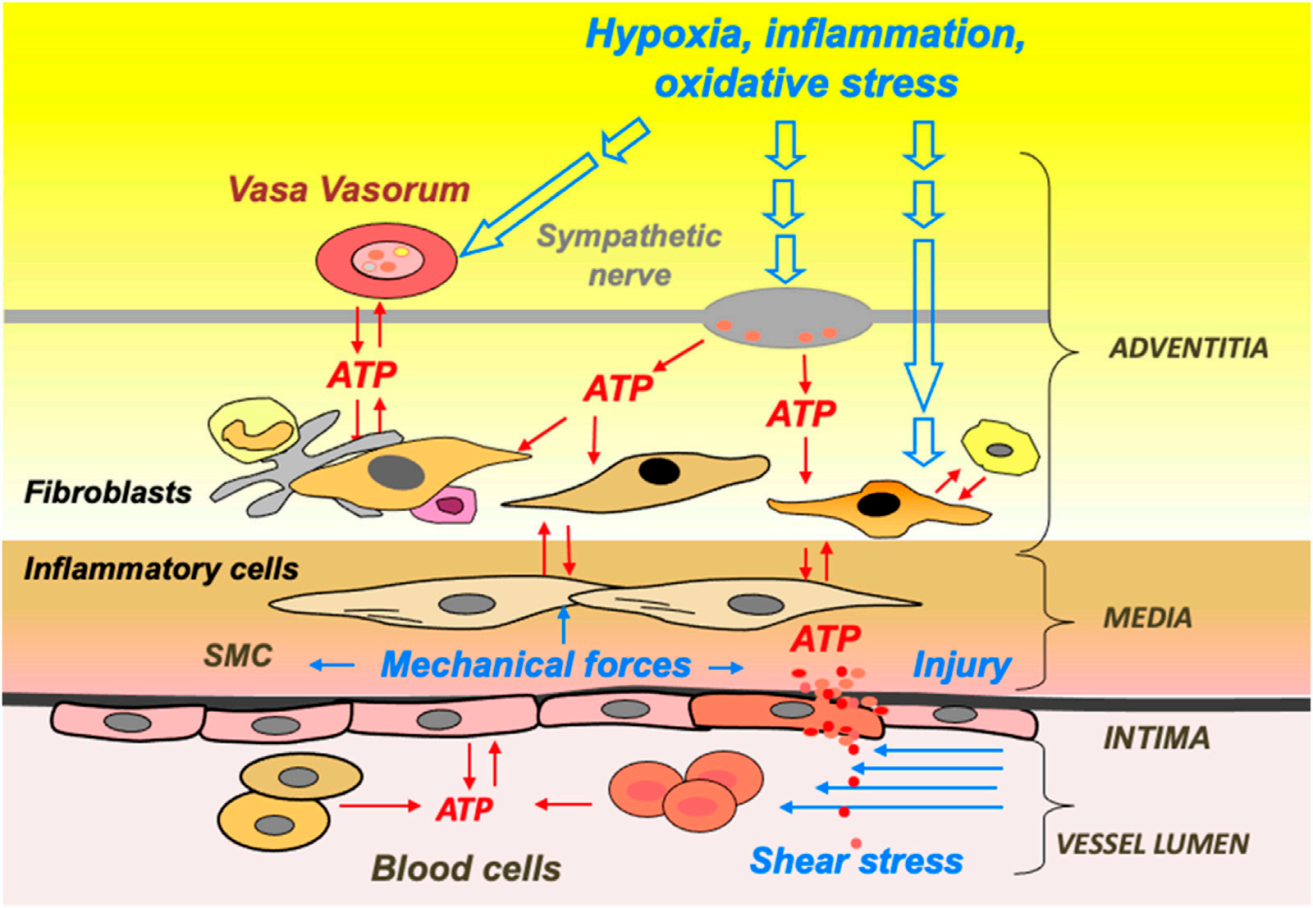
ATP release and signaling are integral to hypoxia- and inflammation-induced vascular remodeling. Schematic presentation of extracellular stimuli and cellular sources of ATP in the vascular wall and lumen.

Extracellular ATP levels are believed to increase within the microenvironment of local tissue in different pathophysiological conditions, such as inflammation, hypoxia, low osmolarity, fluid shear stress, sympathetic stimulation, and thrombosis (Bodin and Burnstock, 1995; Bodin and Burnstock, 2001; Koyama et al., 2001; Gerasimovskaya et al., 2002; Novak, 2003; Idzko et al., 2007; Woodward et al., 2009; Dosch et al., 2018). Additionally, cells within the vasculature, airways, and gut release extracellular nucleotides in response to mechanical forces and other environmental stresses such as changes in osmolarity, hypoxia, hyperoxia, and acidosis, exerting a pivotal function in cellular signaling by activating purinergic receptors within these tissues (Figure 2) (Burnstock, 2002; Lazarowski et al., 2003; Novak, 2003; Woodward et al., 2009; Corriden and Insel, 2010). Moreover, in addition to transiently releasing sub-micromolar or nanomolar concentrations of ATP against various stimuli, cells may also release ATP continuously at basal rates, thereby sustaining pericellular ATP levels within a high micromolar range in the surrounding vicinity (Abbracchio et al., 2006; Yegutkin et al., 2006; Corriden and Insel, 2010). Importantly, this basal release of ATP represents an autocrine and/or paracrine mode of cellular communication and activation provides an economical means for the modulation of cell and tissue biological responses (Corriden and Insel, 2010).

## 3 Human endothelial lung microvascular permeability

### 3.1 Acute lung injury and lung vascular leak

Dating back to the late 1960s, a significant case series published by Ashbaugh et al. (1967) brought attention to acute lung injury (ALI) and acute respiratory distress syndrome (ARDS), albeit not explicitly defined as such at the time. Today, the disease process is characterized by the Berlin definition, published in 2012, supplanting the prior 1994 American-European Consensus definition.

In the United States, the incidence of ALI/ARDS is approximately 78.9–86.2 per 100,000 yearly, or approximately 190,000-200,000 persons per year, with an associated inpatient mortality of 40% (Rubenfeld et al., 2005; Villar et al., 2016). Since 2010, 14 major randomized controlled trials have been published regarding ALI/ARDS adjunctive treatment in mechanically ventilated patients. Interventions including salbutamol, high-frequency oscillation ventilation (HFOV), and lung recruitment with positive end expiratory pressure (PEEP) titration worsened outcomes (Ferguson et al., 2007; Gao Smith et al., 2012; Kacmarek et al., 2016). On the contrary, 12-h daily proning, low tidal volume (TV) with high PEEP, and dexamethasone decreased mortality (Guérin et al., 2013; Villar et al., 2020). A host of other trials, including PEEP titration via esophageal pressure, extracorporeal membrane oxygenation (ECMO), and the use of simvastatin, interferon β-1a, and neuromuscular blockers have not depicted any outcomes (McAuley et al., 2014; Beitler et al., 2019; Moss et al., 2019; Ranieri et al., 2020). Despite the strides made in managing ALI/ARDS, there is substantial growth yet to be done in understanding the pathophysiology of the disease. Simply put, a healthy lung properly regulates fluid across the interstitial and alveolar space. Conversely, a patient whose lungs are compromised with ALI/ARDS has inflammation occurring primarily due to neutrophils as well as endothelial cell damage, both contributing to impaired gas exchange, decreased lung compliance, and pulmonary arterial pressure changes (Wiener-Kronish et al., 1991; Chollet-Martin et al., 1996).

### 3.2 Purine signaling and lung EC barrier

The EC lining the vessels are closely interconnected, thus forming a tight barrier. A prominent feature of ALI/ARDS is a breach in the lung microvascular EC barrier (Kása et al., 2015; Huppert et al., 2019). The data concerning the impact of extracellular purines on the maintenance and alteration of the EC barrier are somewhat conflicting. In human lung microvascular and pulmonary artery EC (HLMVEC and HPAEC, respectively) monolayers, ATP and its derivatives transiently increase transendothelial electrical resistance (TER, an inverse permeability index), indicating EC barrier enhancement (Kolosova et al., 2005; Bátori et al., 2019). On the contrary, it was shown that ATP and its agonists enhanced the paracellular permeability of human umbilical vein EC (HUVEC) (Tanaka et al., 2005) and intact frog and rat microvessels (Pocock et al., 2000; Tanaka et al., 2006). In cultured rat coronary microvascular EC (RCEC), ATP showed a biphasic effect on the permeability of EC monolayers with an initial reduction followed by an increase in permeability to albumin (Noll et al., 1999). ATP may be involved in the formation of inflammasomes by binding to the P2X7 receptor and data from the literature demonstrated that pharmacologic inhibition of the P2X7 receptor exacerbates vascular permeability and inflammation in murine sepsis (Meegan et al., 2022). An antagonist of the P2X7 receptor failed to reverse EC permeability increases in bovine pulmonary microvascular EC induced by an excessively high ATP concentration (5 mM) (McClenahan et al., 2009). Therefore, the involvement of P2X7 receptors in ATP-mediated pulmonary EC barrier regulation requires further investigation. Early work demonstrated that ATP reduces albumin permeability in aortic human EC independently of the rise in intracellular Ca^2+^ (Noll et al., 1999). ATP and its slowly hydrolysable analog, ATPγS, transiently increase TER in the Ca^2+^- and Erk MAP kinase-independent manner with similar maximal effects in HPAEC (Kolosova et al., 2005). Further, a comparison of the effects of ATP and ATPγS on TER of HPAEC revealed similar responses (Kolosova et al., 2005) suggesting a limited role of ATP hydrolysis in ATP-induced human lung EC barrier enhancement. Apparently, the lung EC barrier-enhancing effects of ATP and ATPγS involve the activation of P2Y G-protein coupled receptors (Kolosova et al., 2005; Bátori et al., 2019). It was recently shown that while HLMVEC expressed all known P2Y receptors, only P2Y4 and P2Y12 receptors are involved in ATPγS-induced HLMVEC barrier strengthening presumably via Gi_2_-but not Gs-mediated mechanisms (Bátori et al., 2019). ATP-induced human lung EC barrier enhancement involves Gi_2_-and Gq- but not Gs-coupled signaling (Kolosova et al., 2005). Differences in ATP vs. ATPγS human lung EC signaling are likely due to modest differences in the sensitivity of these agonists to P2 receptors (Burnstock, 2004). Importantly, the barrier-enhancing effects of ATP and ATPγS do not involve Gs trimeric protein activation followed by cAMP increase, but involve activation of protein kinase A (PKA) (Kolosova et al., 2005; Bátori et al., 2019).

Mechanisms of cAMP-independent PKA-dependent EC barrier enhancement in lung EC are not entirely clear, but likely involve the interaction of PKA with a specific scaffolding PKA-binding protein, AKAP2 (Bátori et al., 2019). It was proposed that AKAP2 facilitates the interaction of PKA with myosin light chain (MLC) phosphatase (MLCP) targeting subunit 1 (MYPT1), leading to phosphorylation of MYPT1 on PKA sites, disinhibiting (activating) MLCP, thus, decreasing MLC phosphorylation and opposing contraction (Bátori et al., 2019). Direct involvement of MLCP in ATP and ATPγS-induced human lung EC barrier enhancement was demonstrated (Kim et al., 2012). Further, it was shown that ATP induces MLCP activation/assembly and stimulates dephosphorylation of MYPT1 inhibitory sites in HUVEC (Härtel et al., 2007). Another downstream ATP effector, small GTPase Rac1, tightens EC junctions, at least in part, via cytoskeletal remodeling leading to an increase in the cortical F-actin layer (Jacobson et al., 2006). Rac1 activity has been shown to be directly involved in EC barrier enhancement in HPAEC (Kovacs-Kasa et al., 2018). However, while the interplay between PKA and Rac1 pathways was described in some cells (Bachmann et al., 2013), with Rac1 downstream of PKA in Gs-mediated HUVEC barrier protection (Li et al., 2015), the role (if any) of PKA/Rac1 interplay in ATP-induced lung EC barrier strengthening requires further investigation. Interestingly, cytoskeletal mechanisms of ATP-induced EC barrier enhancement are similar to EC barrier enhancement induced by Sphingosine 1-phosphate, a known platelet-derived EC barrier-enhancing agent (Garcia et al., 2001). Both mechanisms are Gi-dependent and involve the translocation of Rac1 and an actin-binding protein, cortactin, to the cell periphery, suggesting the importance of both proteins in the formation of cortical actin ring (Garcia et al., 2001; Dudek et al., 2004; Jacobson et al., 2006). In addition, it was shown that another actin-binding protein and PKA substrate VASP (vasodilator-stimulated phosphoprotein), was directly involved in ATP-induced increase in TER in HPAEC (Kolosova et al., 2005) thus further supporting the involvement of PKA-mediated cytoskeletal remodeling in ATP-induced EC barrier enhancement. Importantly, ATPγS inhibits the increase in EC permeability induced by lipopolysaccharide (LPS) in HPAEC and protects against LPS and *Escherichia coli*-induced ALI in mice (Kolosova et al., 2008; Gross et al., 2020).

The ultimate purine metabolite of ATP, adenosine, is generated in reaction to metabolic stress and cellular injury. Even, elevated levels of extracellular adenosine are detected in conditions such as inflammation, ischemia, trauma, and hypoxia (Haselton et al., 1993; Umapathy et al., 2010a). Extracellular adenosine transiently enhances human lung macro- and microvascular EC barrier function in cell cultures with about the same magnitude as ATP and ATPγS, but with less duration (Umapathy et al., 2010b; Bátori et al., 2019). On the contrary, opposing effects of ATP and adenosine on EC barrier function were demonstrated in cultured RCEC and rat microvessels (Gündüz et al., 2012), with ATP promoting EC barrier integrity while adenosine weakened the EC barrier. Early work demonstrated that adenosine may be responsible for the apoptotic effects of ATP in HPAEC (Dawicki et al., 1997). Later, it was proposed that while acute adenosine exposure protects from apoptosis via P1 receptor-mediated mechanisms, sustained adenosine exposure caused lung EC apoptosis by the means of nucleoside transporter-mediated intracellular adenosine uptake followed by p38 MAPK and JNK activation in mitochondria thus contributing to cigarette smoke-induced chronic lung diseases (Lu et al., 2013).

However, it was clearly shown that adenosine protects and restores lung vascular barrier integrity in LPS or *E. coli*-induced ALI in murine models (Gonzales et al., 2014; Gross et al., 2020) via a P1 receptor-mediated mechanism (Gonzales et al., 2014). While recent studies demonstrated the involvement of Gs-coupled P1 A2 receptors in lung EC barrier strengthening induced by adenosine, the involvement of specific receptor subtypes (A2A vs. A2B) may be distinct in macro- and microvascular EC due, at least in part, to the difference in the expression level. In HPAEC, it was shown that the expression of A2A and A2B receptors is comparable, but adenosine-induced lung EC barrier enhancement involves only A2A, but not A2B receptors. Comparatively, HLMVEC predominantly expressed A2B receptors with negligible expression of other P1 receptors (Umapathy et al., 2010b; Bátori et al., 2019), and in bovine PAEC (BPAEC), both A2A and A2B receptors are involved in lung EC barrier strengthening (Lu et al., 2010). Interestingly, adenosine promotes EC barrier enhancement in bovine Vasa Vasorum ECs (VVEC) via drastically different mechanisms involving activation of A1 Gi-coupled adenosine receptors, which are predominantly expressed in these cells (Umapathy et al., 2013; Verin et al., 2020). This mechanism is somewhat similar to EC barrier enhancement induced by ATP (or ATPγS) and involves cAMP-independent PKA and Rac1 activation (Verin et al., 2020).

Recent studies on HPAEC and HLMVEC revealed that Gs-mediated signaling involved in adenosine-induced lung EC barrier strengthening involved a rise in cAMP followed by activation of direct cAMP downstream effectors, PKA and Epac1 (Exchange Protein Directly Activated by cAMP 1). Epac one mediates Rac1 activation through the Rap1a/Vav2 but not Tiam1 signaling pathway in HPAEC (Kovacs-Kasa et al., 2018). Depletion of either Rac1, Rap1a, or Vav2, but not Tiam one significantly attenuated the increase in TER induced by adenosine in HPAEC (Kovacs-Kasa et al., 2018). A systematic comparison of the mechanisms of ATPγS and adenosine-induced barrier enhancement in HLMVEC revealed that they both involve PKA activation. In contrast to the ATPγS-mediated mechanism, adenosine-induced PKA activation alone is not sufficient to induce EC barrier enhancement, which requires coordinated activation of both PKA and Epac1 (Bátori et al., 2019). However, both ATPγS- and adenosine-induced barrier enhancements required activation of MLCP downstream of PKA signaling followed by a decrease in MLC phosphorylation (Bátori et al., 2019). Overall, the data suggested that while the mechanisms of ATP and adenosine-mediated human lung EC barrier enhancement are similar on the cytoskeletal level, they are drastically different at upstream receptor-mediated signaling. Precise mechanisms of human lung macro- and microvascular EC barrier strengthening *in vitro* and especially *in vivo* require further investigation.

## 4 Role of purine signaling in vasa vasorum EC

### 4.1 Hypoxia-mediated vasa vasorum angiogenesis and vascular remodeling

Pathological vascular remodeling assumes a crucial role in the advancement of various diseases and conditions characterized by hypoxia, ischemia, or inflammation (Davie et al., 2004; Frid et al., 2006; Shimoda and Laurie, 2013; Gimbrone and García-Cardeña, 2016; Billaud et al., 2018; Jia et al., 2023). Our prior investigation utilizing a neonatal model of pulmonary hypertension revealed substantial alterations in pulmonary vessel structure during the hypertensive process. These changes encompass notable thickening of both the media and adventitia, with particularly remarkable fibroproliferative alterations observed in the adventitia of chronically hypoxic calves (Davie et al., 2004; Stenmark et al., 2006; Nijmeh et al., 2014). Angiogenesis, a phenomenon involving blood vessel expansion can be observed in response to diverse stress conditions affecting microvascular beds, including hypoxia, inflammation, and fibrosis (Carmeliet, 2003; Fong, 2008; Sluimer and Daemen, 2009; Miao et al., 2021). In this context, it is noteworthy that the adventitial compartment of large muscular arteries encompasses a microcirculatory network known as the vasa vasorum, or “vessels of the vessel.” The vasa vasorum sustains vessel integrity by providing oxygen and nutrients to the outer layer of the vessel wall. Recent investigations have underscored that extensive neovascularization of the vasa vasorum might contribute to the progression of vascular diseases in the systemic circulation, including atherosclerosis, ascending aortic aneurysm, acute coronary syndrome, restenosis, and vasculitis (Numano, 2000; Herrmann et al., 2001; Moulton et al., 2003; Hayden and Tyagi, 2004; Choi et al., 2014; Hamaoka-Okamoto et al., 2014; Mulligan-Kehoe and Simons, 2014; Xu et al., 2015; Boyle et al., 2017; Billaud et al., 2018; Sedding et al., 2018; Phillippi, 2022).

In addition, vasa vasorum expansion can be observed in patients with a variety of chronic inflammatory, infectious, and ischemic pulmonary diseases like chronic thromboembolic obstruction, idiopathic pulmonary fibrosis, chronic obstructive pulmonary disease (COPD), and pulmonary hypertension (Charan et al., 1997; Herve and Fadel, 2006; Wagner et al., 2006; McCullagh et al., 2010; Montani et al., 2011; Nijmeh et al., 2014). Extensive neovascularization of the vasa vasorum in the pulmonary artery has been noted in neonatal calves subjected to chronic hypoxia, enabling isolation and culture of vasa vasorum EC representing activated pro-angiogenic phenotype (Burns et al., 2023). Additionally, this neovascularization phenomenon was accompanied by the recruitment of circulating progenitor and inflammatory cells to the pulmonary artery adventitia of calves enduring chronic hypoxia. This suggests that the expanding vasa vasorum serves as both a gateway and conduit for the infiltration and delivery of circulating inflammatory cells into the vessel wall (Davie et al., 2004; Stenmark et al., 2006; Stenmark et al., 2013). These observations may also suggest that the expansion of angiogenic vasa vasorum may represent a common occurrence in pulmonary vascular diseases, with a hypoxic microenvironment potentially facilitating this angiogenic process.

### 4.2 Extracellular ATP as an autocrine regulator of pulmonary artery adventitial fibroblasts and vasa vasorum

While numerous factors with angiogenic potential have been identified, the endogenous molecular factors specifically implicated in the hypoxia-induced expansion of the vasa vasorum, as well as the precise cellular and molecular mechanisms contributing to this process, remain incompletely understood. As previously mentioned, extracellular ATP concentrations can rise in the local tissue microenvironment during various physiological and pathological conditions. Hypoxia has been demonstrated to stimulate the release of ATP from adventitial fibroblasts, and this exogenous ATP may act in an autocrine manner to promote cell proliferation, a response crucial in the vascular remodeling process observed under hypoxic conditions. These findings support the concept that, apart from nerves and circulating blood cells, vascular cells themselves serve as significant sources of ATP and other adenine nucleotides (Gerasimovskaya et al., 2005; Burnstock, 2006; Gerasimovskaya et al., 2008; Woodward et al., 2009).

Studies on isolated pulmonary artery vasa vasorum endothelial cells (VVEC) have indicated ATP release in response to hypoxia suggesting these cells may represent a substantial source of extracellular ATP within the pulmonary artery vascular wall (Burns et al., 2023). It has been demonstrated that ATP release occurs via regulated vesicular exocytosis and involves the activation of PI3K and Rho/ROCK pathways (Van Daele et al., 1992). These observations support the notion that local purinergic signaling networks can be initiated by hypoxic stress, consequently altering endothelial cell phenotype and function. Additionally, given that vascular cells express multiple purinergic receptors, extracellular nucleotides may serve as intercellular signaling molecules facilitating cell-to-cell communication through an autocrine/paracrine mechanism (Bodin and Burnstock, 1995; Bodin and Burnstock, 2001; Koyama et al., 2001; Hisadome et al., 2002; Bours et al., 2006; Gerasimovskaya et al., 2008; Woodward et al., 2009).

### 4.3 Extracellular nucleotide-mediated angiogenic responses in EC

While extracellular purinergic signaling has been associated with numerous physiological and pathological conditions, our understanding of the role of extracellular nucleotides in regulating endothelial cell proliferation remains limited. It has been suggested that EC from different vascular beds display significant functional and morphological diversity (Aird, 2007a), suggesting that the proliferative and angiogenic properties of EC in large vessels and microvessels may vary. Comparative analysis indicated a substantially higher rate of ATP-induced DNA synthesis in pulmonary artery VVEC compared to endothelial cells from the aorta (AOEC), main pulmonary artery (MPAEC), and lung microvascular EC (LMVEC) (Gerasimovskaya et al., 2008). These findings suggest that VVEC isolated from hypoxic or inflammatory environments may possess distinctive phenotypical characteristics with a specific dependence on extracellular nucleotides as an activation stimulus.

Earlier research on EC originating from major vessels revealed that activation of purinergic receptors induces elevation in intracellular calcium levels and the secretion of nitric oxide, t-PA, prostacyclin, and endothelium-dependent hyperpolarizing factor (EDHF) (Motte et al., 1993; Wang et al., 2002). Consistent with these findings, it was observed that ATP and other P2Y receptor agonists induced minimal increases in DNA synthesis in aortic EC (Van Daele et al., 1992; Yamamoto et al., 2003), implying that fully differentiated EC exhibit limited proliferative capacity in response to extracellular nucleotides.

### 4.4 Purinergic receptors, signaling pathways, and transcription factors involved in ATP-induced angiogenic responses in vasa vasorum EC

Drawing from findings in other cell types known for their high proliferative capacity, we focused on the Phosphoinositide 3-kinase (PI3K), mammalian Target of Rapamycin (mTOR), and ERK1/2 pathways. These pathways are pivotal in regulating metastatic cell growth, tumor progression, and angiogenesis (Laplante and Sabatini, 2012; Roskoski, 2012; Thorpe et al., 2015; Fruman et al., 2017; Saxton and Sabatini, 2017). However, evidence linking the activation of these pathways to extracellular ATP and their involvement in angiogenic responses in EC is lacking. Our investigations revealed that extracellular ATP induces angiogenic effects in VVEC by robustly and persistently activating the PI3K/mTOR and ERK1/2 pathways. These responses to extracellular ATP may be particularly significant in the hypoxic and inflamed adventitial microenvironment, where elevated extracellular ATP levels are anticipated. In our recent studies, we observed that unlike VVEC, extracellular ATP had a markedly lesser impact on mTOR, Akt, and ERK1/2 signaling in EC isolated from the pulmonary artery and aorta, resulting in negligible angiogenic effects by ATP in these cells (Gerasimovskaya et al., 2008).

In agreement with exaggerated responses to the extracellular ATP in VVEC, it was demonstrated an expression of wide range of ATP and ADP-sensitive P2Y1, P2Y2, P2Y4, P2Y11, P2Y13, P2Y14, PX2, P2X3, PX4, PX5, PX7 as well as A1, A2B, and A3 adenosine receptors in VVEC isolated from chronically hypoxic animals and control groups (Lyubchenko et al., 2011; Umapathy et al., 2013). It was found that activation of P2Y1, P2Y2, P2Y13, and P2X7 receptors dramatically increased the concentration of intracellular Ca^2+^ (Lyubchenko et al., 2011). The mechanisms of this response involved Ca^2+^ influx through plasma membrane channels and immobilization from intracellular stores. Interestingly, Ca^2+^ influxes were simultaneously observed in both cytosolic and nuclear fractions and Ca^2+^ appeared to rise higher in the nucleus than in the cytoplasm. Using the agonist/antagonist approach, we demonstrated that P2Y1 and P2Y13 receptor-mediated Ca^2+^ responses are functionally linked to activation of ERK1/2, Akt, and S6 pathways, leading to DNA synthesis in VVEC. Importantly, P2Y13R expression has been previously shown to be restricted to circulating progenitor cells, the brain, and the spleen but was not shown in EC. Immunofluorescence analysis revealed co-expression of P2Y13 receptors with CD31, CD34, and CD133 endothelial and progenitor cell markers in the populations of high proliferative VVEC, indicating that hypoxia-induced VV expansion may involve the emergence of endothelial progenitor-like cells expressing P2Y13R (Nijmeh et al., 2014; Strassheim et al., 2020a). Hence, purinergic receptors can be viewed as important signaling components contributing to the activated angiogenic phenotype of VVEC.

TranSignal protein/DNA array in combination with siRNA-mediated knockdown (KD) approach, c-Jun, c-Myc, and Foxo3 were identified as the most sensitive ATP-induced transcription factors in VVEC. In most VVEC populations, these transcription factors were shown to form central nodes connecting several signaling networks. We demonstrated a specific role of these TFs in the regulation of DNA synthesis, migration and tube formation. Additionally, we also showed regulation of angiogenesis-associated target proteins involved in proliferation (Cyclin D, p21^Cip1/Waf1^, stathmin), migration (MMP2, 9, TIMP, PAI-1), tube and cellular junction formation (VE-cadherin, p120 catenin, Connexin 43, ZO-1) (Strassheim et al., 2020b). These findings suggest that pharmacologically targeting the components of the PI3K-Akt-mTOR and Rho-ROCK signaling pathways, as well as specific transcription factors, to reduce hypoxia-induced vasa vasorum expansion may have clinical significance in pathological vascular remodeling in pulmonary hypertension (PH) and possibly other cardiovascular diseases. These diseases include atherosclerosis, restenosis, vasculitis, and aortic aneurysm, which are characterized by enhanced vasa vasorum proliferation (Numano, 2000; Moulton et al., 2003; Hamaoka-Okamoto et al., 2014; Kessler et al., 2014; Sedding et al., 2018).

### 4.5 Extracellular adenosine as a critical regulator of VVEC

Crucially, both extracellular ATP and ADP demonstrate robust vasoactive effects on VVEC, as does extracellular adenosine, which results from the stepwise hydrolysis of ATP by ecto-NTDPase/CD39 and ecto-5′-NT/CD73. The studies have demonstrated that extracellular adenosine, exhibits a barrier protective effect on VVEC. This effect is mediated by the activation of A1 adenosine receptors (A1R) and Gi/PI3K/Akt pathway. It was also found that adaptor proteins, ELMO1, tyrosine phosphatase Shp2, Gαi, and Rac-mediated PKA activation, are involved in VVEC barrier enhancement. This atypical mechanism is not presented in EC of pulmonary circulation indicating a vascular bed-specific effect. However, actin-interacting GTP-binding protein, girdin and the PAK1 downstream target, LIM kinase, are not involved in adenosine-mediated VVEC barrier enhancement. In addition, it was found that adenosine-dependent cytoskeletal rearrangement involved actin severing protein, cofilin dephosphorylation as well as dephosphorylation of cytoskeletal regulatory proteins, ERM (Ezrin, radixin, and moesin) (Umapathy et al., 2013; Verin et al., 2020). Cumulatively, these findings suggest that focusing on A1R and downstream pathways involved in maintaining the barrier integrity of vascular endothelium could offer a novel pharmacological strategy to mitigate hypoxia-induced vascular leakage in pulmonary hypertension (PH) and potentially other cardiovascular diseases characterized by compromised endothelial barrier function.

Several reports demonstrated the involvement of adenosine in endothelial proliferation and chemotaxis, especially under hypoxic conditions, implying adenosine signaling in tissue protection and regeneration (Meininger et al., 1988; Ethier et al., 1993; Linden, 2005; Valls et al., 2009). Concerning adenosine receptor subtypes, research on dermal and retinal EC has indicated the involvement of A2A and A2B receptors, respectively (Grant et al., 2001; Montesinos et al., 2002). Our unpublished data on VVEC demonstrated the mitogenic effects of adenosine and A1 agonists on VVEC similar to what has been observed in the chicken chorioallantoic membrane angiogenic model (Clark et al., 2007). However, the mitogenic effects of adenosine on VVEC appear to be less pronounced compared to ATP and ADP. These findings indicate that adenosine may have a dual effect on VVEC, providing both barrier protection and promoting mitogenesis. This suggests that under normal physiological conditions, adenosine is involved in the normal growth, differentiation, and assembly of intracellular junctions in blood vessels.

## 5 Conclusion and perspectives

We have presented recent updates demonstrating the significance of extracellular purines as autocrine-paracrine signaling molecules, influencing lung vascular pathologic responses, angiogenesis, and permeability regulation. Conditions such as chronic hypoxia and inflammation can alter extracellular nucleotide concentrations within the vascular wall due to injurious stimuli and regulated ATP release. This process integrates extracellular ATP and its hydrolysis products into purinergic signaling networks. The endothelial-specific molecular mechanisms of ATP release their regulation by inflammatory cytokines and hypoxic stress remain not fully explored and require a more detailed investigation. In addition, systematic exploration of vascular bed-specific profiles of purinergic receptors and ecto-nucleotidases help better understanding endothelial phenotypic diversity in relation to physiological and pathological responses.

Given the recent insights into the pivotal role of purinergic signaling in pulmonary diseases, a significant gap is the comprehensive understanding of how chronic versus acute hypoxia and inflammation distinctly impacts purine metabolism. There is a pressing need to delve deeper into the differential effects of chronic and acute hypoxia on extracellular and intracellular purine metabolism to uncover novel interconnections between regulatory enzymes and networks that may be at play.

Additionally, innovative transcriptomic approaches, such as single-cell RNA sequencing, are essential for identifying previously unknown genes and pathways regulated by purinergic signaling. The application of various bioinformatic platforms will aid in understanding the integration of the components of purinergic signaling pathways into the hierarchy of complex pathways that govern cellular phenotype and function under normal and pathologic conditions. These innovative approaches will also facilitate the elucidation of how hypoxia and other injurious stimuli affects purinergic intercellular communication within the vascular microenvironment.

Exploring pharmacological strategies to modulate purinergic signaling pathways as potential therapies for diseases characterized by pathological hypoxia is crucial. For instance, treatment with selective P2Y receptor antagonists in combination with P1 receptor agonists may mitigate excessive angiogenesis while promoting endothelial barrier improvement. Moving forward, continued advancements in purinergic pharmacology field will enrich our understanding of the intricate role of extracellular purines in vascular biology, potentially paving the way for therapeutic strategies targeting purinergic signaling in vessel injury, growth, and maintenance.
